# Answers to burning questions for clinical allergologists related to the new COVID-19 vaccines

**DOI:** 10.1007/s40629-021-00177-3

**Published:** 2021-07-14

**Authors:** Sabine Altrichter, Stefan Wöhrl, Fritz Horak, Marco Idzko, Galateja Jordakieva, Eva Untersmayr, Zsolt Szepfalusi, Petra Zieglmayer, Erika Jensen-Jarolim, Ursula Wiedermann, Alexander Rosenkranz, Wolfram Hötzenecker

**Affiliations:** 1 Department of Dermatology und Venerology, Comprehensive Allergy Center, Kepler University Hospital, Krankenhausstraße 9, 4020 Linz, Austria; 2 Department of Dermatology und Allergology, Charité—Universitätsmedizin Berlin, Berlin, Germany; 3 Floridsdorfer Allergiezentrum (FAZ), Vienna, Austria; 4 Allergiezentrum Wien West, Vienna, Austria; 5 Department for Medicine II, Pulmonology, Medical University of Vienna, Vienna, Austria; 6 Department of Physical Medicine, Rehabilitation and Occupational Medicine, Medical University of Vienna, Vienna, Austria; 7 Department of Pathophysiology und Allergy Research, Center for Pathophysiology, Infectiology and Immunology, Medical University of Vienna, Vienna, Austria; 8 Department of Pediatrics and Adolescent Medicine, Division for Pediatric Pulmonology, Allergology and Endokrinology, Comprehensive Center for Pediatrics, Medical University of Vienna, Vienna, Austria; 9 Competence Center for Allergology and Immunology, Karl Landsteiner Privatuniversität für Gesundheitswissenschaften, Krems, Austria; 10 Department of Interdisciplinary Life Sciences, Messerli Research Institute, University of Veterinary Medicine, Vienna, Austria; 11 Institute of Specific Prophylaxis and Tropical Medicine, Center for Pathophysiology, Infectiology and Immunology, Medical University of Vienna, Vienna, Austria; 12 Division of Nephrology, Department for Medicine, Medical University Graz, Graz, Austria

**Keywords:** COVID-19, Allergy, Anaphylaxis, Vaccination, SARS-CoV‑2

## Abstract

**Background:**

Along with the newly approved vaccines against coronavirus disease 2019 (COVID-19), first reports of allergic or intolerance reactions were published. Subsequently, questions arose whether these vaccines pose an increased risk for intolerance reactions and whether allergic patients may be at higher risk for this.

**Results:**

Allergic reactions following COVID-19 vaccinations have been reported, but mostly of mild severity and at normal (Moderna®) or only slightly increased frequency (BioNTech/Pfizer®) compared to established conventional vaccines. The risk of allergic reaction to the newly licensed vector vaccines (AstraZeneca®, Johnson&Johnson®) cannot be conclusively assessed yet, but also appears to be low. There is currently no evidence that patients with allergic diseases (atopic patients) react more frequently or more severely to these vaccines. It is currently assumed that intolerance reactions of the immediate-type are either type I allergic (IgE-mediated) reactions or occur via complement activation (CARPA, “complement activation-related pseudoallergy”). Polyethylene glycol (PEG) or polysorbate, which are present as stabilizers in the vaccines, are suspected as triggers for this.

**Conclusion:**

The data available so far do not show a significantly increased risk of immediate-type allergic reactions in atopic persons. In almost all cases, atopic patients can be vaccinated without problems. Standardized follow-up tests after suspected allergic reactions or CARPA-mediated reactions are currently limited.

## Vaccinations against COVID-19 from the allergist’s point of view

According to the WHO (World Health Organization), 63 vaccines are currently in clinical trials [1]. As of March 2021, four vaccines have been approved in the European Union (EU) and three are already being vaccinated: first, Comirnaty® from Pfizer/BioNTech [2] and mRNA-1273 from Moderna® have been approved. Both preparations are novel mRNA-based (mRNA, “messenger RNA”) vaccines in which viral mRNA is encapsulated in lipid nanoparticles [3]. Accordingly, both vaccines contain additives (Table 1). One additive (ALC-0159) contains a polyethylene glycol (PEG) polymer with a molecular weight of about 2000 g/mol (PEG 2000), which is an intermediate-sized molecule (compared with the PEG lengths used in a variety of cosmetics and drugs as an additive or in PEGylated drugs [from 300 to about 40,000 g/mol]). Adjuvants are not included in these vaccines, nor are preservatives or hen’s egg protein.

**Table 1:** Composition of COVID-19 vaccines submitted for approval or already approved (post-EUA) in the EU by March 2021. (Modified from [25])

Vaccine, manufacturer ((7))	Ingredients	Status
BNT162b2 (Comirnaty®), Pfizer/BioNTech	mRNA ALC-0315 ((4-hydroxybutyl)azanediyl)bis(hexane‑6,1‑diyl)bis(2-hexyldecanoate) ALC-0159 (2-[(**polyethylene glycol**)-2000]-N,N-ditetradecylacetamide) 1,2-Distearoyl-sn-glycero-3-phophocholine Cholesterol Potassium chloride, Potassium dihydrogen phosphate, Sodium chloride, Disodium hydrogen phosphate-dihydrate, Sucrose Multi-use bottle: natural latex	Post-EUA
mRNA-1273 (Moderna®), Moderna Biotech	mRNA SM (sphyngomyelin)-102 **Polyethylenglycol [PEG] 2000** dimyristoyl glycerol [DMG] 1,2-distearoyl-sn-glycero-3-phosphocholine [DPSC] Cholesterol Tromethamine hydrochloride, Acetic acid, Sodium acetate, Saccharose	Post-EUA
AZD1222 (Vaxzevria®/AstraZeneca), AstraZeneca	modifiedVirus (ChAdOx1 nCoV-19) **Polysorbate 80** **=** **Polyoxyethylene-20-sorbitan monooleate** Histidine, Saccharose, Sodium chloride, Magnesium chloride, Sodium EDTA, Ethanol	Post-EUA
Ad26.COV2.S (Janssen), Johnson & Johnson	Adenivirus26 vectored vaccine Sodium chloride, Citric acid-monohydrate **Polysorbate 80** **=** **Polyoxyethylene-20-sorbitan monooleate** 2‑hydroxylpropyl-B-cyclodextrin (HBCD) Ethanol, Sodium hydroxide	Post-EUA
NVX-CoV2373, Novavax	Recombinant SARS-CoV‑2 glycoprotein nanoparticle with MatrixM adjuvant **Polysorbate 80** **=** **Polyoxyethylene-20-sorbitan monooleate** Matrix M1 adjuvant	Phase III

*EU* European Union, *EUA* emergency use authorization, *mRNA* messenger-RNA

The other approved vaccines are vector-based vaccines from AstraZeneca and Johnson & Johnson. These vaccines do not contain PEG 2000, but do contain a structurally related molecule (polysorbate 80 = polyoxyethylene-20-sorbitan monooleate). Currently, other vaccines are currently tested in phase III trials, which also contain polysorbate 80 (Table 1).

Type I allergic, IgE-mediated reactions have been reported in single cases to (mostly long-chain) PEG of various sizes [4, 5]. Immediate-type allergic reactions to PEG have been described in individual cases, for example, after bowel cleansing in preparation for colonoscopy in which patients were administered a large amount of PEG. Since PEG is included as an additive in a variety of products (laxatives, intravenous drugs such as cortisone), it is also suspected as a “hidden” allergen [6–8]. Possible type IV sensitization to (predominantly short-chain) PEGs (e.g., PEG 400) from prior use of cosmetics or “dermal fillers” [9] containing PEGs is also possible. PEGs are also used in dental products (bleachings, toothpastes). Apart from this, pseudoallergic (non-IgE-mediated) reactions (so-called CARPA, “complement activation-related pseudoallergy”) have also been described after contact with liposomes [3, 10, 11]. These reactions are attributed in part to the binding of pre-existing anti-PEG IgM to liposomes with subsequent complement activation. Clinical symptoms of this non-IgE-mediated hypersensitivity have been described including dyspnea, tachypnea, hypotension and hypertension shortly after intravenous administration of liposome-containing drugs. Independent of possible PEGylation, liposomes themselves have been attributed to activate complement nonspecifically as well (depending on their different surface structures and charge) [3].

**Fig. 1:**
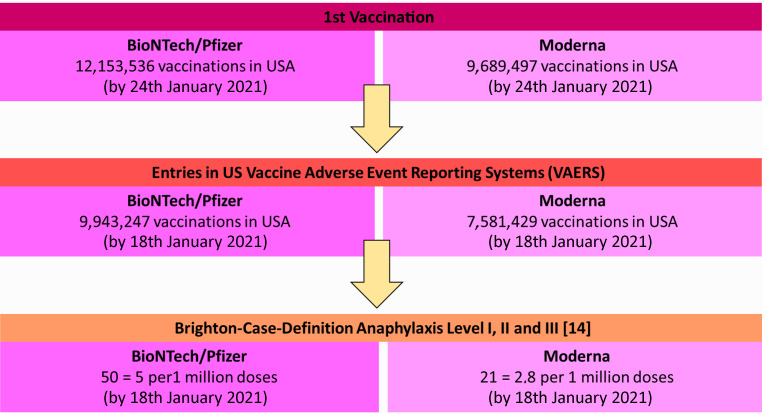
Summary of allergic reactions published to date after COVID-19 mRNA vaccination. (As of 12 February 2021; modified from [13])

So far recorded side effects after COVID-19 mRNA vaccination were local reactions, fever, serum sickness or other side effects (exanthema, pain and redness at the vaccination site, myalgia, temperature elevation, etc.). These common reactions are also observed after other vaccinations and should not be considered as allergic or pseudoallergic reaction to the vaccine. Previously reported cases of anaphylactic reactions after COVID-19 mRNA vaccination (Fig. 1) have been overall rare, with 2.5 incidents per million initial doses administered for the mRNA1273 vaccine (Moderna®). Slightly higher rates have occurred upon the use of Comirnaty® vaccine (Pfizer/BioNTech) (5 per 1 million initial vaccinations) [12, 13]. All allergic reactions reported to date that were rated as Brighton safety grade I or II (very high or high diagnostic certainty of an allergic/anaphylactic reaction; [14]) have had mild reactions such as skin symptoms (such as redness, itching, whealing) or skin reactions and pulmonary symptoms (such as shortness of breath, cough, asthma, stridor, hypoxia). None of the vaccinees required hospitalization after initial emergency treatment. Interestingly, 90% of the patients with the previously described reactions were women. The reactions occurred mostly within 15 min, but in individual patients as late as up to 2.5 h after vaccination. Whether these observed reactions were due to IgE-mediated or IgE-independent pseudoallergic (possibly CARPA) reactions is unknown, as of yet. Similarly, it is unclear whether these reactions were triggered by the vaccine per se or by the additives, such as PEG 2000.

The reported deaths that occurred after COVID-19 vaccination are most likely not due to allergic reactions to the vaccine, but can be explained in part by the advanced age of the first vaccination target group in nursing homes (senile age, multiple comorbid diseases, etc.) or by specific side effects of the vaccines [13].

Currently, there are no published scientifically analyzed review articles on allergic reactions to the vaccines from AstraZeneca and Johnson & Johnson. Anaphylaxis after vaccination with AstraZeneca vaccine has been reported. The European Medicines Agency (EMA) has recorded 535 cases related to 22 million vaccinated AstraZeneca doses, but these have been reported by both medical and nonmedical personnel and have not yet been subject to an allergological workup. Accordingly, the resulting theoretical number of 22 cases per million doses includes very likely a high number of vaccine adverse events that do not meet the criteria for anaphylaxis [15]. For the Johnson & Johnson vaccine, two anaphylactic reactions have been reported in the media to date [16]. There have been no official written reports or statements regarding these events up to date (as of 17 March 2021). In the published clinical trials using the Johnson & Johnson vaccine in 40,000 subjects, no anaphylactic reaction was reported [17].

## COVID-19 vaccination in patients with allergic diseases

Based on the data from studies up to date, it can be concluded that for patients with allergic diseases (e.g., rhinoconjunctivitis allergica, bronchial asthma, food allergies, insect venom allergies, drug allergies), there is no increased likelihood of an anaphylactic reaction following COVID-19 vaccination [18]. One-third of the cases with allergic reactions observed had no known prior allergic disease [12]. The majority of cases in which reactions occurred within minutes after vaccination were diagnosed as nonimmunologic reactions (such as vasovagal syncope). In order to achieve information about the pathomechanism in cases of presumed anaphylactic reactions, blood sampling can be used to determine serum mast cell tryptase and complement blood levels (C3/C4/CH50) immediately after emergency treatment and patient stabilization [19]. Allergies, other than known immediate-type allergy to a component of the vaccine, are therefore not a problem for COVID-19 vaccination, based on current data.

**Fig. 2:**
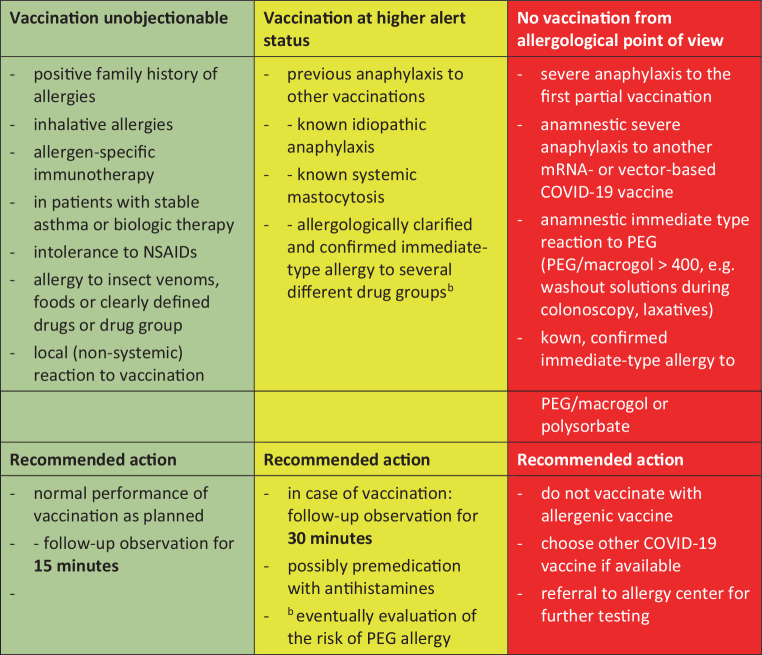
Procedure for patients with known allergic or atopic diseases; Date: 12 February 2021. *mRNA* messenger-RNA, *NSAR* non-steroidal anti-inflammatory drug, *PEG* Polyethylenglycol. (© AG Allergologie der ÖGDV)

Patients with previous anaphylaxis to vaccination, known idiopathic anaphylaxis, known systemic mastocytosis, or with confirmed allergies to a wide variety of drugs from different groups, may take premedication (antihistamines) before vaccination (Fig. 2). In addition, prolonged follow-up of 30 min may be considered. In general, vaccination centers should be staffed with medical personnel who can administer emergency treatment for potentially severe reactions. Emergency medications including epinephrine pens, infusion and intubation equipment should be kept on hand, and staff must be trained in resuscitation.

## Interactions of COVID-19 vaccinations and existing therapies in patients

During specific immunotherapy (subcutaneous or sublingual allergen immunotherapy [AIT]), patients taking antiallergic medications (antihistamines, leukotriene antagonists, topical and systemic glucocorticoids) or receiving therapy with biologics for their allergic disease (e.g., omalizumab, dupilumab, benralizumab, mepolizumab, reslizumab) can receive the vaccination [20]. It is generally recommended that these therapies (AIT, biologics) should be given one to two weeks before or after the COVID-19 vaccination [21]. Sublingual immunotherapy (SLIT) can continue to be taken as usual. A break on the day of COVID-19 vaccination and possibly one to two days afterwards may be recommended to distinguish possible vaccine-related side effects from reactions to the SLIT.

Vaccination may also be given during immunosuppressive systemic therapies (high-dose glucocorticoids, cyclosporine A, methotrexate). There are no data to date that have shown poorer tolerability of vaccination while using these therapies. However, comparable to other vaccines/inactivated-vaccines, it can be assumed that maybe only reduced or inadequate vaccine protection is established while under immunosuppression.

## Possibilities and limitations of allergological workup

Currently, it is recommended that persons who have reacted to the first vaccination with a severe, possibly allergic reaction or a presumably anaphylactic shock shall undergo an allergological evaluation and clarification prior to a possible second COVID-19 vaccination. Unfortunately, the additives used in the vaccinations are often not available for allergy testing. In addition, data on the sensitivity and specificity of these test substances in skin tests (e.g., skin prick test or intradermal test) or cellular test approaches (e.g., basophil activation test [BAT], cellular stimulation test [CAST]) are lacking. PEG with different molecular weights can be obtained as a pure substance and, like polysorbate 80, may be used for “in house” prick tests. Such tests had be used in case reports for the clarification of an immediate-type allergy to PEG or polysorbate. In general, cross-reactivity between PEG and polysorbates is likely to be underestimated, and might increase in direct proportion to the size of the PEG [6], and could be IgE-mediated [5]. However, whether these tests allow an estimation of the allergological risk in the context of COVID-19 vaccination is unknown. Direct testing with the vaccine is currently not possible. On one hand, the vaccine shortage would not allow such testing from an ethical point of view and, on the other hand, no type of testing and no test concentrations or test reactivity with the vaccine are known or established. Contact allergies to PEG have been described and are also regularly recorded in epicutaneous testing (using PEG400). In the few known cases where different PEGs were tested, only low cross-reactivity between low molecular weight PEG allergy and high molecular weight PEG (PEG4000 or PEG3350) allergy was found [22]. To date, there has been no reported case of allergic reaction after COVID-19 vaccination in patients with known type IV sensitization to PEG.

## Discussion

According to the current knowledge, allergic or anaphylactic reactions to the novel COVID-19 vaccines are very rare, but up to approximately ten times more frequent than in conventional vaccines. Patients with known atopies and allergic diseases have no increased risk of an allergic reaction to the vaccination. Lipid nanoparticles within the vaccines, and especially the PEG contained therein, is suspected as triggering agent for hypersensitivity reactions. In vector vaccines (AstraZeneca and Johnson & Johnson vaccines), polysorbate 80 is a possible allergen. Testing procedures that allow the prediction of (pseudo-﻿)allergic intolerance reactions to COVID-19 vaccinations or exclude them are only available to a very limited extent. Allergic and intolerance reactions can manifest with clinically severe (anaphylactoid) symptoms and usually occur within 15–30 min after vaccine administration. Personnel and technical equipment for emergency treatment are therefore necessary in vaccination centers. Allergic persons or persons with a history of anaphylaxis have no contraindication for vaccination. However, a history of allergic reactions to vaccine ingredients (e.g., PEG) is currently a contraindication to vaccination, as well as an anaphylactic reaction to the first dose of a COVID-19 vaccine, which is a contraindication to administration of the second dose of the same preparation. In agreement with the Center for Disease Control [23], the authors recommend that regardless of the vaccine-induced antibody titer, the second vaccination should be withheld until valid data are available supporting a second vaccination with an alternative preparation.

Apart from anaphylaxis on first injection, which presupposes pre-existing sensitization [24], no statement can currently be made as to whether the first partial vaccination per se can lead to specific sensitization. A total of only four cases of anaphylactic reaction after the second administration have been documented [13].

According to the European product information, a safety follow-up of a minimum of 15- to 30-minutes post vaccination should be performed in all vaccinated individuals. Appropriate medical treatment and monitoring should always be available, in case a severe allergic intolerance reaction after vaccine administration occurs.

## Abbreviations

AIT: Allergen immunotherapy
BAT: Basophil activation test
CARPA: Complement activation-related pseudoallergy
CAST: Cellular stimulation test
COVID: Corona virus disease
MRNA: Messenger RNA
PEG: Polyethylene glycol
SLIT: Sublingual immunotherapy
